# The acid–base and redox properties of menaquinone MK-4, MK-7, and MK-9 (vitamin K_2_) in DMPC monolayers on mercury

**DOI:** 10.1007/s00249-020-01433-0

**Published:** 2020-05-05

**Authors:** Karuppasamy Dharmaraj, Javier Ignacio Román Silva, Heike Kahlert, Uwe Lendeckel, Fritz Scholz

**Affiliations:** 1 Institute of Biochemistry, University of Greifswald, Felix-Hausdorff-Str. 4, 17487 Greifswald, Germany; 2 Institute of Medical Biochemistry and Molecular Biology, University Medicine Greifswald, University of Greifswald, Ferdinand-Sauerbruch-Str., 17475 Greifswald, Germany

**Keywords:** Menaquinones, Acidity constants, Standard potentials, Lipid monolayer, DMPC, Electrochemistry

## Abstract

**Abstract:**

The acid–base and redox properties of the menaquinones MK-4, MK-7, and MK-9 (vitamin K_2_) have been studied in DMPC monolayers on mercury electrodes. The monolayers were prepared by adhesion-spreading of menaquinone-spiked DMPC liposomes on a stationary mercury drop electrode. All three menaquinones possess ${\text{p}}K_{{\text{a}}}$ constants outside the experimentally accessible range, i.e., they are higher than about 12. The standard potentials of MK-4, MK-7, and MK-9 in the DMPC monolayers are very similar, i.e., 0.351, 0.326, and 0.330 V (corresponding to the biochemical standard potentials − 0.063, − 0.088, and − 0.085 V).

**Graphic abstract:**

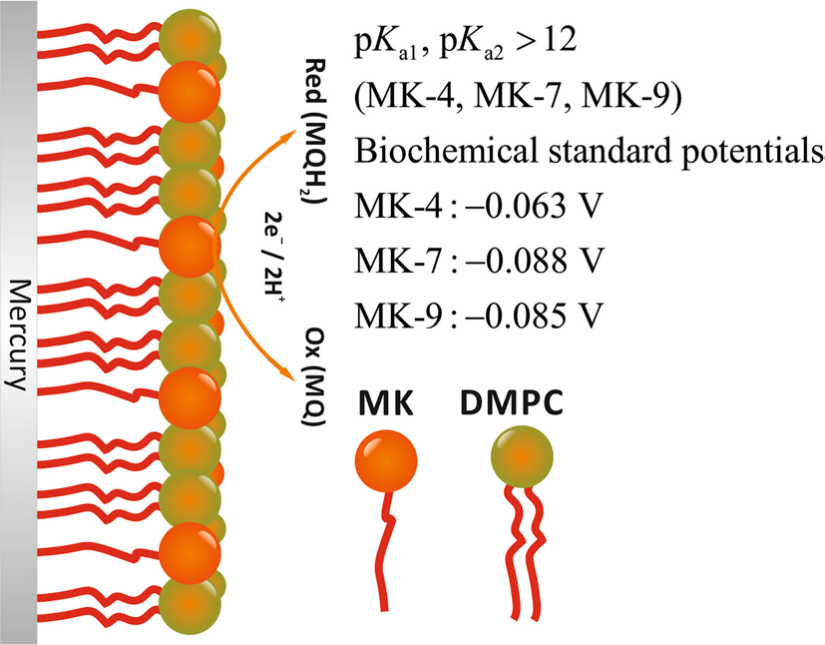

## Introduction

A very recent review of the electrochemistry of vitamins highlights the importance of solid thermodynamic data, and it also shows that no reliable data concerning Vitamin K are available (Lovander et al. 2018). Vitamin K, in its hydroquinone state, functions as an exclusive coenzyme of γ-glutamyl carboxylase (GGCX, EC 4.1.1.90), which catalyzes the post-translational γ-carboxylation of a number of vitamin K-dependent proteins (Kleuser 2018). Thereby, important physiological and pathophysiological processes such as blood coagulation, bone metabolism, arterial calcification, oxidative stress, and extrahepatic tissue energy metabolism are regulated (Chatron et al. 2019). Vitamin K is not a single compound but represents a multitude of chemically related molecules with similar biological activity. All K vitamins possess a 2-methyl-1,4-naphthoquinone moiety (menadione, vitamin K_3_). In menaquinones (K_2_ vitamins), substituents are present in position 3 consisting of different numbers of isoprenyl units (MK-1 to MK-14). GGCX is only activated by vitamin K in its hydromenaquinone state, which in this reaction is converted to the inactive vitamin K epoxide (Oldenburg et al. 2008). Its reactivation is mediated by vitamin K epoxide reductase (VKORC1) (Oldenburg et al. 2008). Studying the interaction of menaquinones, menadione, and phyllochinones with VKORC1, Chatron and co-workers revealed that indeed the length of the isoprenoid substituent determines the affinity of vitamin K derivatives to the VKORC1 (Chatron et al. 2019): binding free energy of the epoxide forms to VKORC1 was highest for MK-7, followed by vitamin K_1_ and MK4, whereas that of K_3_ (lacking any side chain) was by far lower. This distinguished feature of MK-7 is in accordance with the beneficial effects of this particular derivative in preventing vascular and bone diseases, decreasing the risk of cancer (Nimptsch et al. 2008) and diabetes (Beulens et al. 2010), and decreasing the risk of coronary artery diseases in dialysis patients (Gast et al. 2009). As to how these different biological activities of menaquinones with different isoprenoid chain length is due to their distinct acid–base and redox properties remains to be elucidated.

The compounds studied in this work include the prominent K_2_ vitamin family members MK-4, MK-7 as well as a more lipophilic derivate, MK-9. They are listed in Table 1. The biological functions, the biosynthesis and dietary aspects of menaquinones are well covered in the literature (Shearer et al. 2008; Maklashina et al. 2006; Cotrim 2016; Gröber et al. 2014) and do not need to be discussed here.

**Table 1:**
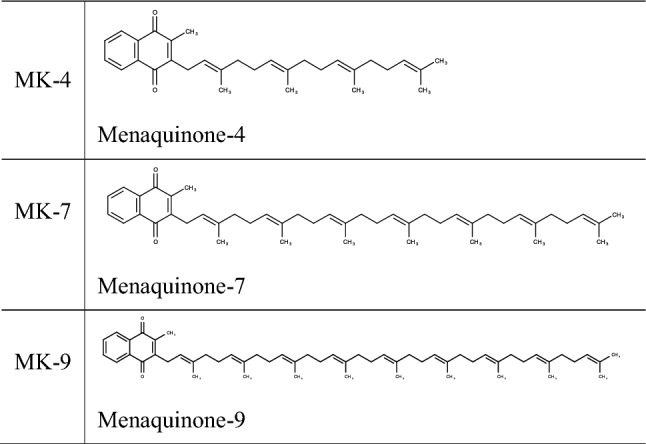
The three menaquinones used in this study

The menaquinones listed in Table 1 were incorporated in DMPC (1,2-dimyristoyl-sn-glycero-3-phosphocholine) monolayers on the surface of mercury electrodes (stationary hanging drops) to study their redox and acid–base equilibria. The DMPC molecules of the monolayer on mercury form an ‘ordered fur’ of molecules with the polar phosphatidylcholine head groups facing the water interface. It is probable, but not yet proven that the menaquinone molecules are arranged between the DMPC molecules with the naphthoquinone head groups also facing the water interface. Whereas MK-4 has a C_16_ chain like the two palmitoyl chains of DMPC, MK-7 has a C_28_ and MK-9 even a C_36_ chain. Clearly, MK-7 and MK-9 have to assume a bended structure, either partly sandwiched between the DMPC monolayer and the mercury surface, or bended between the DMPC molecules.

When the oxidized form is abbreviated by MQ, and the reduced by MQH_2_, the following overall equation describes the coupled redox and acid–base equilibria:

Equilibrium I $${\text{MQ } + \text{ 2e}}^{ - } + \text{ 2H}_{3} {\text{O}}^{ + } \, \rightleftarrows {\text{ MQH}}_{2} + \text{ 2H}_{2} {\text{O}}$$

This equilibrium can be split as follows, in the pure redox equilibrium.

Equilibrium II $${\text{MQ } + \text{ 2e}}^{ - } \, \rightleftarrows {\text{ MQ}}^{2 - }$$

having the standard potential $E_{{{\text{MQ/MQ}}^{2 - } }}^{{\ominus }}$ and the two acid–base equilibria.

Equilibrium III $${\text{MQH}}_{2} + \text{ H}_{2} {\text{O }} \rightleftarrows {\text{ MQH}}^{ - }  + \text{ H}_{3} {\text{O}}^{ + } \, $$

Equilibrium IV $${\text{MQH}}^{ - } + \text{ H}_{2} {\text{O }} \rightleftarrows {\text{ MQ}}^{2 - } + \text{ H}_{3} {\text{O}}^{ + }$$

having the two acidity constants $K_{{{\text{a1}}}}$ and $K_{{{\text{a2}}}}$ (or ${\text{p}}K_{{{\text{a1}}}}$, and ${\text{p}}K_{{{\text{a2}}}}$, resp.). The reduction of MQ can proceed in two one-electron steps with a semiquinone radical as intermediate, which may also exist in two protonated forms (see Aguilar-Martínez et al. 2000, where the electrochemistry of 2-phenylamin-1, 4-naphthoquinone in acetonitrile is presented). However, in an aqueous environment, the semiquinones are normally unstable.

The pH dependence of Equilibrium I is described by the following equation (Scholz and Kahlert 2019):

1 $$ E_{{{\text{MQ/MQ}}^{2 - } }} = E_{{{\text{MQ/MQ}}^{2 - } }}^{{\ominus}} + \frac{RT}{{2F}}\ln \frac{{a_{{{\text{MQ}}}} }}{{a{}_{{{\rm{MQ}}^{2 - } }}}} + \frac{RT}{{2F}}\ln \left( {\frac{{a_{{{\text{H}}_{3} {\rm{O}}^{ + } }}^{2} }}{{K_{{{\text{a1}}}} K_{{{\rm{a2}}}} }} + \frac{{a_{{{\text{H}}_{3} {\rm{O}}^{ + } }} }}{{K_{{{\text{a2}}}} }} + 1} \right) $$

Hence, the formal potential $E_{{{\text{c, MQ/MQ}}^{2 - } }}^{{\ominus ^{\prime}}}$, defined for $a_{{{\text{MQ}}}} = a_{{{\text{MQ}}^{2 - } }}$, is:

2 $$ E_{{{\text{c, MQ/MQ}}^{2 - } }}^{{\ominus ^{\prime}}} = E_{{{\text{MQ/MQ}}^{2 - } }}^{{\ominus }} + \frac{RT}{{2F}}\ln \left( {\frac{{a_{{{\text{H}}_{3} {\rm{O}}^{ + } }}^{2} }}{{K_{{{\text{a1}}}} K_{{{\text{a2}}}} }} + \frac{{a_{{{\text{H}}_{3} {\rm{O}}^{ + } }} }}{{K_{{{\text{a2}}}} }} + 1} \right) $$

The constants $E_{{{\text{MQ/MQ}}^{2 - } }}^{{\ominus }}$, ${\text{p}}K_{{{\text{a1}}}}$, and ${\text{p}}K_{{{\text{a2}}}}$ can be experimentally determined by plotting the formal potentials versus pH and fitting the plot with Eq. (2), provided there is a pH region in which the formal potentials are independent of pH. The ${\text{p}}K_{{\text{a}}}$ values can only be determined if they are positioned in the accessible pH range, i.e., between 0 and 14. In case of the menaquinones, the insolubility of these compounds in aqueous solutions is a serious problem for electrochemical measurements. A very early developed strategy to cope with this problem was the preparation of thin films of the insoluble compounds on solid electrodes (Ksenzhek et al. 1977; Petrova et al. 2000). Although, this allowed electrochemical measurements, the state of the molecules in the solid film remains unknown and the meaning of the determined data remains unclear. Later, solid quinoide compounds, esp. ubiquinones, have been incorporated into lipid films on solid electrodes (Gordillo et al. 2000; Marchal et al. 1997) which to a much better extent approaches the situation of these compounds in real membranes. Recently, we have shown that lipid monolayers containing ubiquinones (the coenzymes Q_10_ and Q_4_) can be prepared on stationary mercury electrodes (Heise and Scholz 2017), via the adhesion-spreading of quinone-spiked liposomes (Hellberg et al. 2002, 2005; Agmo Hernández et al. 2008, 2013). The same strategy has been followed here to prepare DMPC monolayers spiked with menaquinones in order to allow cyclic voltammetric measurements from which the mid-peak potentials have been taken as the formal potentials (under the provision that the mid-peak potentials do not seriously deviate from the formal potentials) (Scholz 2010).

Standard potentials and acidity constants of compounds dissolved in water are strictly defined for all involved species in the solvated (hydrated) state. Clearly, this is not the case for molecules in a lipid film. Hence, these data should be labelled ‘formal acidity constants’ and ‘formal potentials’ in the respective films. They are more akin to similar data of acids and redox species confined to a solid surface. Further, since they are part of a dielectric layer on a metal electrode, effects of the hydrophobic environment have to be considered (White et al. 1998; Pashkovskaya et al. 2018) (see “Results and discussions”). Another complication may also arise from the mobility of the immobilized compounds in the film, i.e., the mobility of the lipid molecules and the menaquinones. The latter may change their position when charged and deprotonated, as it has been discussed for carbonic acids (Creager and Clarke 1994).

## Experimental section

### Chemicals

The following chemicals were used: citric acid (analytical grade) was from Serva Feinbiochemica GmbH, Germany, trisodium citrate pentahydrate (extra pure) was from Laborchemie, Apolda GmbH, Germany, disodium monohydrogen phosphate dihydrate (Na_2_HPO_4_·2H_2_O) (≥ 98%), sodium hydroxide (NaOH) (≥ 99%), potassium chloride (KCl) (≥ 99.5%), chloroform (HPLC grade), and methanol (≥ 99.98%, ultra LC–MS grade) were from Carl Roth GmbH, Germany, monosodium dihydrogen phosphate dihydrate (NaH_2_PO_4_·2H_2_O) (pure pharma grade) was from Applichem GmbH, Germany, disodium carbonate monohydrate (Na_2_CO_3_·H_2_O) (> 99%) was from Fluka Chemika, Germany, sodium bicarbonate (NaHCO_3_) was from Merck, Germany, DMPC (14:0 PC) (1,2-dimyristoyl-sn-glycero-3-phosphocholine) lipid was from Avanti Polar Lipids, USA, menaquinone 4 (MK-4) and menaquinone 7 (MK-7) were from Sigma Aldrich, Germany; menaquinone 9 (MK-9) was from Caymann Chemical, Germany. The buffer solutions were prepared using citric acid/trisodium citrate pentahydrate for pH 4, Na_2_HPO_4_·2H_2_O/NaH_2_PO_4_·2H_2_O for pH 6 and 7.4, Na_2_CO_3_·H_2_O/NaHCO_3_ for pH 9, Na_2_HPO_4_·2H_2_O/NaOH for pH 11 and 11.4 and NaOH for pH 12–14.

### Instrumentation

Cyclic voltammograms were recorded with an AUTOLAB PGSTAT 12 (Metrohm, Switzerland) in conjunction with the electrode stand VA 663 (Metrohm, Switzerland). A multimode electrode in the Hanging Mercury Drop Electrode (HMDE) mode (drop size 2, surface area 0.464 mm^2^) served as working electrode, a platinum rod and an Ag|AgCl (3 M KCl, *E *= 0.207 V vs. SHE) were used as auxiliary and reference electrodes, respectively. The surface area of the mercury drops has been determined via weighing 6 times 50 mercury drops and calculating the surface area assuming complete sphericity. The standard deviation of the surface area data was 0.0065 mm^2^. The redox systems were studied with cyclic voltammetry (staircase) in normal mode using the scan rates 10, 25, 50, 100, 200 mV s^−1^ and a step potential of 0.00412 V. A temperature-controlled bath (Lauda Ecoline 003 E100) was used to ensure that all measurements were performed at 25 °C. All the experiments were repeated at least three times and the mean values were used for the calculations. Chronocoulometry was performed with 10 cycles keeping the electrode in each cycle for 5 s at $E_{{{\text{ox}}}} = E_{{{\text{midpeak}}}} + 100{\text{ mV}}$ and at $E_{{{\text{red}}}} = E_{{{\text{midpeak}}}} - 100{\text{ mV}}$. The interval time was 0.1 s. The pH meter (Qph70, VWR) was calibrated using the buffer solutions pH 2.00 (± 0.02), pH 7.96 (± 0.02) from Carl Roth, Germany, and pH 12.00 (± 0.05) from VWR, Germany. The pH measurements were conducted for all buffer solutions and for pH 14 which was realised by using 1 mol L^−1^ NaOH solution. This solution provides also strong buffering because of its high hydroxide concentration. The number of MK molecules ($n_{{{\text{MK}}}}$) was calculated by summing up the surface areas occupied by MK ($S_{{{\text{MK}}}}$) and DMPC ($S_{{{\text{DMPC}}}}$) molecules and relating the sum to the surface area of the hanging mercury drop ($S_{{{\text{mercury}}}}$): $S_{{{\text{mercury}}}}  = S_{{{\text{DMPC}}}} { + }S_{{{\text{MK}}}}$, $S_{{{\text{mercury}}}} { = ( }n_{{{\text{DMPC}}}} \times S_{{{\text{DMPC}}}}^{*} {) + ( }n_{{{\text{MK}}}} \times S_{{{\text{MK}}}}^{*} {)}$, $n_{{{\text{DMPC}}}} = r \times n_{{{\text{MK}}}}$, $n_{{{\text{DMPC}}}}$: number of DMPC molecules, $n_{{{\text{MK}}}}$: number of MK molecules, $r$: ratio of the numbers of molecules of DMPC to MK, $S^{*}_{{{\text{DMPC}}}}$: surface area of one DMPC molecule, $S^{*}_{{{\text{MK}}}} \,$: surface area of one MK molecule.

### Liposome preparation

Liposomes were prepared according to the modified rapid evaporation method developed by Moscho group (Moscho et al. 1996). The DMPC lipids were dissolved using chloroform, methanol and the desired amounts of menaquinone (for the final concentrations: 2 µmol L^−1^, 5 µmol L^−1^, 10 µmol L^−1^ MK) were added from a chloroform stock solution (1 mg mL^−1^). Final concentrations of 300 µmol L^−1^ DMPC containing menaquinone (2 µmol L^−1^, or 5 µmol L^−1^, or 10 µmol L^−1^ of MK-4 or MK-7 or MK-9) were obtained by adding 20 mL of buffer pH 7.4.

### Preparation of lipid monolayers on mercury electrode

The liposome suspension was deaerated at least for 30 min with nitrogen. A new mercury drop was formed and then the solution was stirred for 15 min. Then the solution was exchanged with the required buffer for studying the redox properties and the solution was purged with nitrogen for 30 (± 2) minutes. The solution exchange is mandatory to avoid any possible response caused by suspended liposomes.

## Results and discussion

**Fig. 1:**
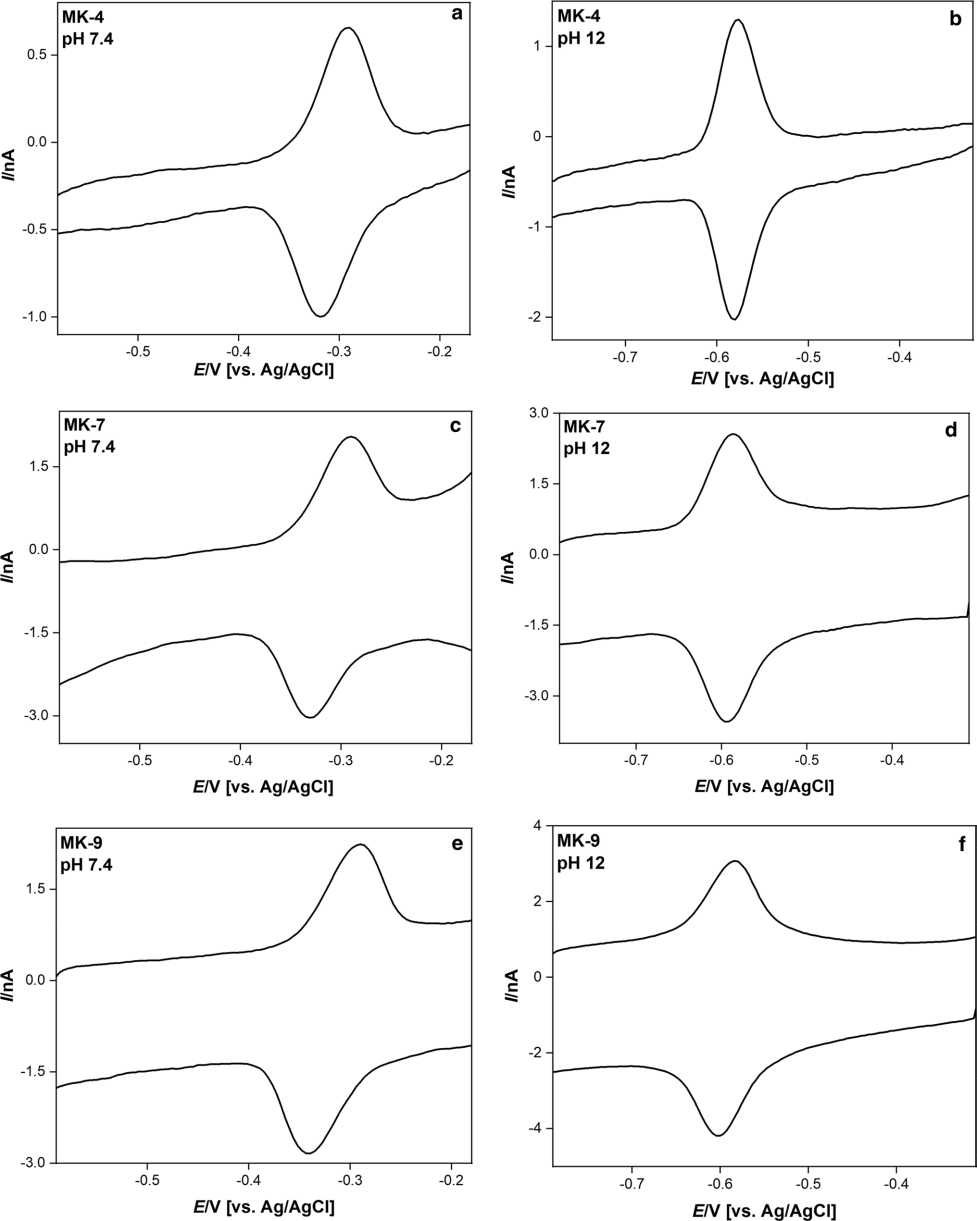
Cyclic voltommograms of DMPC films spiked with MK-4, -7, and -9 at pH 7.4 and 12. The film composition was 300 µmol DMPC + 2 µmol menaquinones. The scan rate was 25 mV s^−1^

The cyclic voltammograms of DMPC films spiked with MK-4, -7 and -9 at pH 7.4 and 12 are shown in Fig. 1.

Table 2 lists the peak separations at the scan rate of 10 mV s^−1^. The separation of anodic and cathodic peaks is generally small, with an even decreasing tendency at pH values larger than 11. The small peak separation is typical for immobilized redox systems (thin-layer behaviour).

**Table 2:** Separation of anodic and cathodic peaks for DMPC films spiked with MK-4, -7, and -9

pH	Peak separation [mV]		
	MK-4	MK-7	MK-9
4.0	8 (± 2)	10 (± 2)	10 (± 2)
6.0	22 (± 11)	22 (± 4)	23 (± 6)
7.4	18 (± 7)	26 (± 2)	33 (± 0)
9.0	15 (± 2)	23 (± 8)	14 (± 2)
11.0	10 (± 2)	6 (± 2)	10 (± 2)
12.0	4 (± 3)	4 (± 0)	8 (± 4)
12.4	8 (± 0)	5 (± 2)	8 (± 0)
13.0	11 (± 2)	3 (± 5)	4 (± 0)
14.0	6 (± 4)	6 (± 4)	8 (± 0)

The film composition was 300 µmol DMPC + 2 µmol menaquinones. The scan rate was 10 mV s^−1^. In brackets, the standard deviations are given, which are based on at least three measurements

The mid-peak potentials at constant pH are almost constant in the range of 10 to 200 mV s^−1^, just being scattered within ± 5 mV, and do not differ significantly for all three menaquinones (*p* = 0.05).

**Fig. 2:**
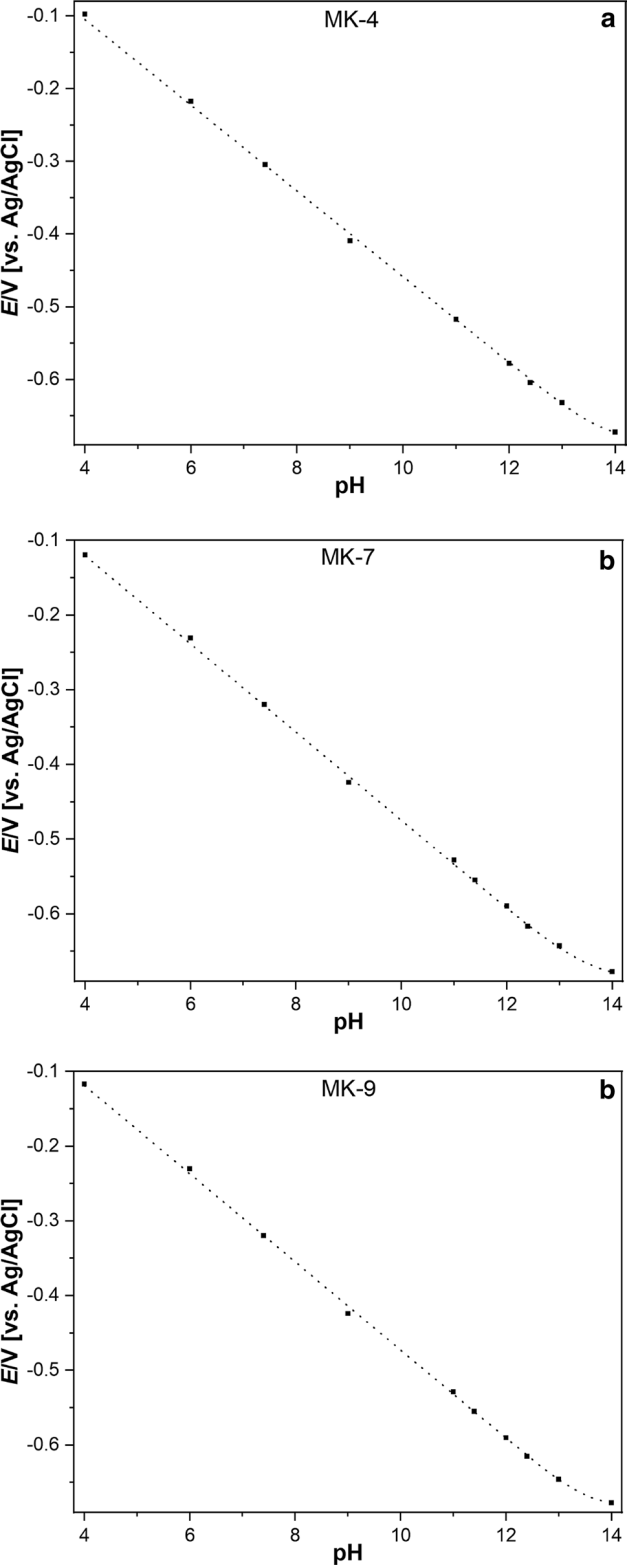
Dependence of mid-peak potentials of cyclic voltammograms of the menaquinone spiked DMPC films on pH

Between pH 4 and 12, the mid-peak potentials obey strictly linear dependences (the slopes are given in Table 3), and above pH 12 the curves are bent towards much smaller slopes. This bending can have two reasons: (i) if the two ${\text{p}}K_{{\text{a}}}$ values are smaller than pH 14, the slope would be about 30 mV in the range ${\text{p}}K_{{{\text{a1}}}} {\text{ < pH < p}}K_{{{\text{a2}}}}$, and zero for ${\text{p}}K_{{{\text{a2}}}} {\text{ } < \text{ pH}}$ (cf. Eq. (2)). (ii) Another reason for the bending could be the interference of another cation, e.g., sodium ions, because pH values above 11 were realised with sodium hydroxide. Then the bending can happen because sodium ions are bond by the anionic species of the hydroquinone forming ${\text{MQNa}}^{ - }$ and ${\text{MQNa}}_{2}$, respectively. This case would resemble the sodium interference of the glass electrode response. To decide about the reasons of the bending, experiments have been performed by keeping the pH constant and changing the sodium concentration. At sodium concentrations of 0.5 to 2 mol L^−1^ the mid-peak potentials are slightly increasing, but the pH dependence is still almost linear for pH smaller than 11. This suggests that the ${\text{p}}K_{{\text{a}}}$ values are most probably really above 12, but does not exclude an interference by sodium. At so large sodium concentration (0.5 to 2 mol L^−1^) activity coefficients deviate so much from unity that a data interpretation is excluded. The least square fitting of the dependences shown in Fig. 2 with Eq. (2) produced in all cases (no significant difference at *p* = 0.05) identical ${\text{p}}K_{{{\text{a1}}}}$ and ${\text{p}}K_{{{\text{a2}}}}$ values of 13.7 (± 1.3). The only solid conclusion which can be drawn from the results is that the ${\text{p}}K_{{\text{a}}}$ values are for sure higher than 12 and a sodium response is, if at all, very weak.

**Table 3:** Slopes of mid-peak potentials versus pH functions of DMPC films spiked with MK-4, -7, and -9 in the pH range 4 to 11

Menaquinones	Slopes [mV/pH]
MK-4	− 60.63 (± 1.00)
MK-7	− 59.30 (± 1.32)
MK-9	− 59.70 (± 1.29)

The film composition was 300 µmol DMPC + 2 µmol menaquinones. In brackets, the standard deviations are given, which are based on at least 45 measurements

The fitting of the dependences shown in Fig. 2 is based on assuming that the number of electrons is two. To test this assumption, coulometric measurements have been performed. This is not an easy task, because for coulometric measurements, the number of menaquinone molecules on the electrode surface has to be known and was determined as described in the experimental part.

The liposomes have been prepared with varying ratios of DMPC to MK-4 molecules (from 300:1 to 30:1). For the DMPC and MQ molecules rod-like (cylindrical) geometries have been assumed. The base area of the DMPC cylinder was taken as $60.6 \cdot 10^{ - 20}$ m^2^ and that of MK-4 $34.1 \cdot 10^{ - 20}$ m^2^. According to Heppes (Heppes 2003), the maximum surface coverage for two-size disc packing is 0.91. With these data, the results given in Table 4 were obtained. With increasing dilution, the number of electrons approaches 2. This is in remarkable good agreement with the assumption of a $2{\text{e}}^{ - } /2{\text{H}}^{ + }$ process, given the many possibilities of errors (e.g., weighing the compounds, liposome formation, film formation, packing geometry, molecule geometry).

**Table 4:** Number of electrons transferred between the reduced and oxidized states of MK-4, as determined in coulometric experiments at different ratios of MK-4: DMPC

MK-4: DMPC	No. of electrons in two separate measurements
1:300	1.96 2.37
1:200	2.11 2.13
1:150	1.81 1.89
1:100	2.11 1.78
1:60	2.20 0.98
1:30	1.24 2.5

At least 2 different monolayers were studied for each ratio. The mean number of electrons for all measurements was 1.92

Although the above mentioned identical values of ${\text{p}}K_{{{\text{a1}}}}$ and ${\text{p}}K_{{{\text{a2}}}}$, resulting from the least-square fitting, cannot be taken as strictly proven, this would not be surprising: the phenomenon of identical ${\text{p}}K_{{{\text{a1}}}}$ and ${\text{p}}K_{{{\text{a2}}}}$ data of immobilized quinoide compounds is well known (Ksenzhek et al. 1977; Masheter et al. 2007; Lee et al. 2013). Identical ${\text{p}}K_{{\text{a,i}}}$ values have been observed for drop casted films on graphite electrodes, adsorbed quinones, as well as for covalently bond hydroquinones. Anthraquinone modified carbon nanotubes on graphite have also two indistinguishable ${\text{p}}K_{{\text{a,i}}}$ values of 13, and even greater than 14. The authors concluded that different molecular environments and electronic coupling determine the dissociation constants (Masheter et al. 2007). In addition, adsorbed mercaptohydroquinone on gold has larger ${\text{p}}K_{{\text{a,i}}}$ values than in aqueous buffer solution (Sato et al. 1996). However, when these compounds are dissolved in solution, the two values ${\text{p}}K_{{{\text{a1}}}}$ and ${\text{p}}K_{{{\text{a2}}}}$ are well separated. In Table 5 are listed the ${\text{p}}K_{{{\text{a1}}}}$ and ${\text{p}}K_{{{\text{a2}}}}$ data of quinoide compounds dissolved in aqueous solutions.

**Table 5:** ${\text{p}}K_{{{\text{a1}}}}$ and ${\text{p}}K_{{{\text{a2}}}}$ data of hydroquinones in aqueous solutions

	${\text{p}}K_{{{\text{a1}}}}$	${\text{p}}K_{{{\text{a2}}}}$	${\Delta p}K_{1,2}$	Ref
1,4-Benzohydroquinone	9.9 9.91	11.9 12.04	2 2.13	Bailey and Ritchie (1985) Abichandani and Jatkar (1938)
1,4-Naphtohydroquinone	9.3	11.2	1.9	Bailey and Ritchie (1985)
1,4-Anthrahydroquinone (Aqueous solution containing 5%DMF)	10 9	12 12.05	2 3.05	Masheter et al. (2007) Revenga et al. (1994)
2-Methyl-napthohydroquinone	10.4	12.55	2.15	Ksenzhek et al. (1977)
2-Methyl-napthohydroquinone	11.5	12.5	1.0	Driebergen et al. (1990)

The merging of the two ${\text{p}}K_{{\text{a,i}}}$ values is just one typical feature of immobilized dibasic acids. The other is a remarkable increase of these values compared to the data of acids dissolved in solutions (cf. Table 6). The increase of the ${\text{p}}K_{{\text{a,i}}}$ values of immobilized acids is generally between 2 and 5 units (White et al. 1998; Creager et al. 1994; Chechik et al. 2000), which equals to 11.4 to 29 kJ mol^−1^.

**Table 6:** ${\text{p}}K_{{\text{a}}}$ values of some carboxylic acids, thiophenol and mercaptopyridine immobilized on surfaces and dissolved in solutions

	Surface immobilized acid ${\text{p}}K_{{\text{a}}}$	Acid dissolved in solution ${\text{p}}K_{{\text{a}}}$	References
4-Mercaptopyridine	4.6	1.4	Bryant and Crooks (1993)
4-Aminothiophenol	6.9	4.3	
HS(CH_2_)_2_COOH	6.5–8.4	4.3	Burris et al. (2008)
HS(CH_2_)_15_COOH	8.0, 6.4	4.5	Chechik et al. (2000)
HS(CH_2_)_10_COOH	5.5–8.5	4.5	
HS(CH_2_)_7_COOH	8.0	4.5	
HS(CH_2_)_5_COOH	6.0	4.5	
HS(CH_2_)_2_COOH	5.8, 8.0	4.5	

So far, the increase of ${\text{p}}K_{{\text{a,i}}}$ values has been ascribed to the hydrophobic environment of the acidic groups in the films. However, we think that this is not convincing, as it is well known that the permittivity of the medium and its donor–acceptor properties with respect to protons are most important (Izutsu 1990). Especially for covalently bonded hydroquinones, it is most likely that the acid groups are exposed to the aqueous phase and not housed in a hydrophobic pocket. A “hydrophobic environment” would also be unable to explain the merging of the two ${\text{p}}K_{{\text{a,i}}}$ values, as in organic solvents the ${\text{p}}K_{{\text{a,i}}}$ values are usually well separated. For dissolved dibasic acids with completely independent protonation sides, Adams has shown already in 1916, that the ratio of the two acidity constants cannot be smaller than $K_{{{\text{a1}}}} :K_{{{\text{a2}}}} = 4$ (Adams 1916). This ratio was indeed observed in several cases of dissolved acids.

The above-described dependence of mid-peak potentials of cyclic voltammograms of the menaquinone spiked DMPC films on pH is in general agreement with the observations made by other authors in case of immobilized dibasic acids (Masheter et al. 2007; Lee et al. 2013). As discussed before, these authors interpreted their behavior using the common model of two deprotonation steps characterized by the two constants ${\text{p}}K_{{{\text{a1}}}}$ and ${\text{p}}K_{{{\text{a2}}}}$, and finding that these constants are equal, i.e., ${\text{p}}K_{{{\text{a1}}}} = {\text{p}}K_{{{\text{a2}}}}$. This, however, allows writing just one acid–base equilibrium:

Equilibrium V $${\text{MQH}}_{2} {\text{ } + \text{ 2H}}_{2} {\text{O }} \rightleftarrows {\text{ MQ}}^{2 - } {\text{ } + \text{ 2H}}_{3} {\text{O}}^{ + } \, $$

in conjunction with the redox equilibrium.

Equilibrium II $${\text{MQ } + \text{ 2e}}^{ - } \, \rightleftarrows {\text{ MQ}}^{2 - }$$

The two-proton acid–base equilibrium has the following equilibrium constant:

3 $$ K_{{{\overline{\text{a}}}}} = \frac{{a_{{{\text{MQ}}^{2 - } }} a_{{{\text{H}}_{3} {\text{O}}^{ + } }}^{2} }}{{a_{{{\text{MQH}}_{2} }} }} = K_{{{\text{a1}}}} K_{{{\text{a2}}}} $$

The Nernst equation for Equilibrium II is:

4 $$ E_{{{\text{MQ/MQ}}^{2 - } }} = E_{{{\text{MQ/MQ}}^{2 - } }}^{{\ominus }} + \frac{RT}{{2F}}\ln \frac{{a_{{{\text{MQ}}}} }}{{a{}_{{{\text{MQ}}^{2 - } }}}} $$

5 $$ E_{{{\text{MQ/MQ}}^{2 - } }} = E_{{{\text{MQ/MQ}}^{2 - } }}^{{\ominus }} - \frac{RT}{{2F}}\ln K_{{{\overline{\text{a}}}}} + \frac{RT}{F}\ln a_{{{\text{H}}_{3} {\text{O}}^{ + } }}^{{}} + \frac{RT}{{2F}}\ln \frac{{a_{{{\text{MQ}}}} }}{{a_{{{\text{MQH}}_{2} }} }} $$

Since ${\text{p}}K_{{{\text{a1}}}}$ and ${\text{p}}K_{{{\text{a2}}}}$ are larger than 11 or 12, and not exactly accessible, only the standard potential $E_{{{\text{MQ/MQH}}_{2} }}^{{\ominus }}$, i.e., the standard potential relating to the couple ${\text{MQ / MQH}}_{2}$, can be extracted from the experimental dependences shown in Fig. 2. The results are given in Table 7.

We propose the following explanation of the behaviour of immobilized dibasic acids:

i. From a thermodynamic point of view, an immobilized acid is not anymore an individual molecule, but it is a separate phase with many acidic groups on its surface, possessing a distribution of ${\text{p}}K_{{\text{a}}}$ values, resulting in one average ${\text{p}}K_{{\text{a}}}$ value. Because the overall equilibrium involves two electrons and two protons (for aqueous solutions adjacent to the film containing the immobilized acids) it makes sense to define one ${\text{p}}K_{{{\overline{\text{a}}}}}$ value referring to a two-proton equilibrium. Whether the ${\text{p}}K_{{{\overline{\text{a}}}}}$ in Eq. (3) is the product $K_{{{\text{a1}}}} K_{{{\text{a2}}}}$ or resulting from a distribution of many individual ${\text{p}}K_{{\text{a}}}$ values cannot be decided now.
ii. The thermodynamics of the protolysis of immobilized acids also fundamentally differs from that of dissolved acids by producing an immobile, i.e., fixed, anion. It is well established that the protolysis of dissolved carbonic acids is mainly driven by the entropy of the formed ions ${\text{B}}^{ - }$ and ${\text{H}}_{3} {\text{O}}^{ + }$ from ${\text{HB}}$, i.e., by the structuring of the solvent around these ions (Sarmini and Kenndler 1999; Calder and Barton 1971). The enthalpy changes are rather small. The decrease of entropy gain caused by the fixation of the anion must inevitably lead to a higher stability of the protonated form ${\text{HB}}$, i.e., a lower acidity (larger ${\text{p}}K_{{\text{a}}}$ value). Clearly, the free energy of protolysis is dominated by the contribution from proton solvation (Liptak and Shields 2001).

The formal potentials of MK-4, -7, -9 at different surface concentrations (2 µmol, 5 µmol, 10 µmol per 300 µmol DMPC) are practically constant. When the concentration is increased above 20 µmol per 300 µmol DMPC, the redox systems behave very differently, possibly because of phase transitions and formation of separate domains of DMPC and menaquinone. This will be studied in near future.

## Conclusions

The menaquinones MK-4, MK-7, and MK-9 possess in DMPC monolayers on mercury very similar standard potentials (cf. Table 7). Applying the two ${\text{p}}K_{{\text{a,i}}}$ model, the three menaquinones have practically identical acidities, and for each menaquinone both ${\text{p}}K_{{\text{a,i}}}$ values are indistinguishable so that the acidity can also be characterized by one ${\text{p}}K_{{{\overline{\text{a}}}}}$ value for a two-proton step. The experimentally found identity of the two ${\text{p}}K_{{\text{a,i}}}$ values does not mean that they are really identical: ${\text{p}}K_{{\text{a,i}}}$ values between 13 and 14 imply a rather large uncertainty. The acidity constants make clear that under any physiological conditions, only the completely protonated forms exist. Both the ${\text{p}}K_{{\text{a,i}}}$ values and the standard potentials determined in this study make it understandable that these compounds can act as highly efficient molecules to transfer two electrons and two protons in one step.

**Table 7:** Standard redox potentials $E_{{{\text{MQ/MQH}}_{2} }}^{{\ominus }}$ and biochemical standard potentials $E_{{{\text{c, MQ/MQH}}_{2} }}^{{\ominus ^{\prime}}}$ of the menaquinones in a DMPC layer, as derived from the plots given in Fig. 2

Menaquinones	Standard redox potential $E_{{{\text{MQ/MQ}}^{2 - } }}^{{\ominus }}$, (V) vs SHE	Biochemical standard potential $E_{{{\text{c, MQ/MQ}}^{2 - } }}^{{\ominus ^{\prime}}}$, (V) vs SHE
MK-4	0.351	− 0.063
MK-7	0.326	− 0.088
MK-9	0.330	− 0.085

## Data Availability

Primary data are stored at the University of Greifswald.
